# Lower-profile stent graft reduces the risk of embolism during thoracic endovascular aortic repair in shaggy aorta

**DOI:** 10.1093/icvts/ivad058

**Published:** 2023-04-24

**Authors:** Yoshimasa Seike, Kenta Masada, Tetsuya Fukuda, Koki Yokawa, Shigeki Koizumi, Mio Kasai, Yosuke Inoue, Hiroaki Sasaki, Hitoshi Matsuda

**Affiliations:** Department of Cardiovascular Surgery, National Cerebral and Cardiovascular Center, Osaka, Japan; Department of Cardiovascular Surgery, National Cerebral and Cardiovascular Center, Osaka, Japan; Department of Radiology, National Cerebral and Cardiovascular Center, Osaka, Japan; Department of Cardiovascular Surgery, National Cerebral and Cardiovascular Center, Osaka, Japan; Department of Cardiovascular Surgery, National Cerebral and Cardiovascular Center, Osaka, Japan; Department of Cardiovascular Surgery, National Cerebral and Cardiovascular Center, Osaka, Japan; Department of Cardiovascular Surgery, National Cerebral and Cardiovascular Center, Osaka, Japan; Department of Cardiovascular Surgery, National Cerebral and Cardiovascular Center, Osaka, Japan; Department of Cardiovascular Surgery, National Cerebral and Cardiovascular Center, Osaka, Japan

**Keywords:** Aortic arch aneurysm, Descending thoracic aortic aneurysm, Thoracic endovascular aortic repair, Low-profile stent graft, Embolism, Stroke, Spinal cord injury

## Abstract

**OBJECTIVES:**

This study aimed to reveal the association between lower-profile stent graft (LPSG) and embolism during thoracic endovascular aortic repair for non-dissecting distal arch and descending thoracic aortic aneurysm.

**METHODS:**

This study reviewed data of 35 patients who underwent thoracic endovascular aortic repair with LPSG (27 males; age: 77 ± 9.2 years) and 312 who underwent thoracic endovascular aortic repair with conventional-sized stent graft (CSSG) (247 males; age: 77 ± 7.4 years) from 2009 to 2021.

**RESULTS:**

The rate of total embolic events was significantly lower in the LPSG group (0/35 [0%]) than the CSSG group (34/312 [11.2%]) (*P *=* *0.035). Shaggy aorta (odds ratio: 5.220; *P *<* *0.001) were identified as positive embolic event predictors. The rate of total embolic events in 68 patients with shaggy aorta (12 in LPSG/56 in CSSG) was significantly lower in the LPSG group (0/12 [0%]) than the CSSG group (19/56 [34%]) (*P *=* *0.015). The rate of total embolic events in 279 patients with the non-shaggy aorta (23 in LPSG/256 in CSSG) reveals no difference between the 2 groups (0 [0%]/16 [6.3%]) (*P *=* *0.377).

**CONCLUSIONS:**

LPSG usage could reduce embolism in thoracic endovascular aortic repair, and the difference was more pronounced in patients with the shaggy aorta. LPSG might be beneficial in preventing embolism in thoracic endovascular aortic repair for patients with a shaggy aorta.

**Figure:**
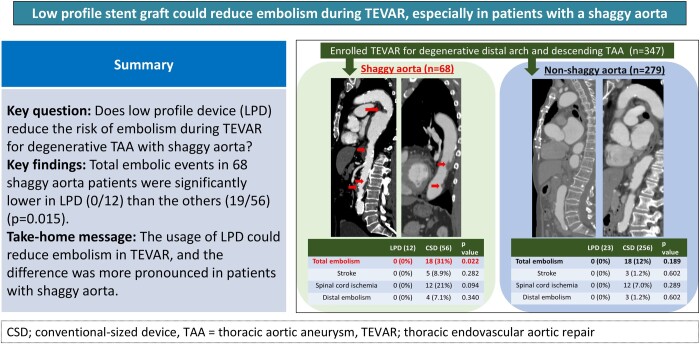

## INTRODUCTION

Thoracic endovascular aortic repair (TEVAR) for degenerative distal arch and descending thoracic aortic aneurysms (TAA) is generally the first-line procedure focusing on elderly patients but is also widely used for all age groups. However, applying TEVAR in patients with a severe atheromatous change in the aorta, also known as ‘shaggy aorta’, remained debateable due to a considerable complication of embolism [1, 2].

Management, including the use of balloon protection for the target vessels, filter devices and trapping emboli in an arterial filter, has been reported to prevent embolic events [3–6]. However, these measures are methods to retrieve embolized materials, but not to prevent embolization itself. Furthermore, the device and its procedure itself can be invasive and cause embolism.

Fundamentally, embolism prevention would be ideal, of which possible mechanism includes the scratching of the aorta by the stent graft, and reduced friction between the stent graft and the aortic wall may reduce embolism itself. Among recent advancements in TEVAR technology, lower-profile stent graft (LPSG) might be effective in reducing friction during TEVAR compared with conventional-sized devices [7]. This study aimed to reveal the association between LPSG and embolism during TEVAR for non-dissecting distal arch and descending TAA.

## MATERIALS AND METHODS

### Ethics statement

This observational study was officially approved by the Institutional Review Board (M30-036). The need for individual oral and written informed consent was waved due to the retrospective design of the study. Instead of individual consent, we began obtaining the subject's information after confirming the posting of the opt-out document.

### Study design and study population

**Figure 1:**
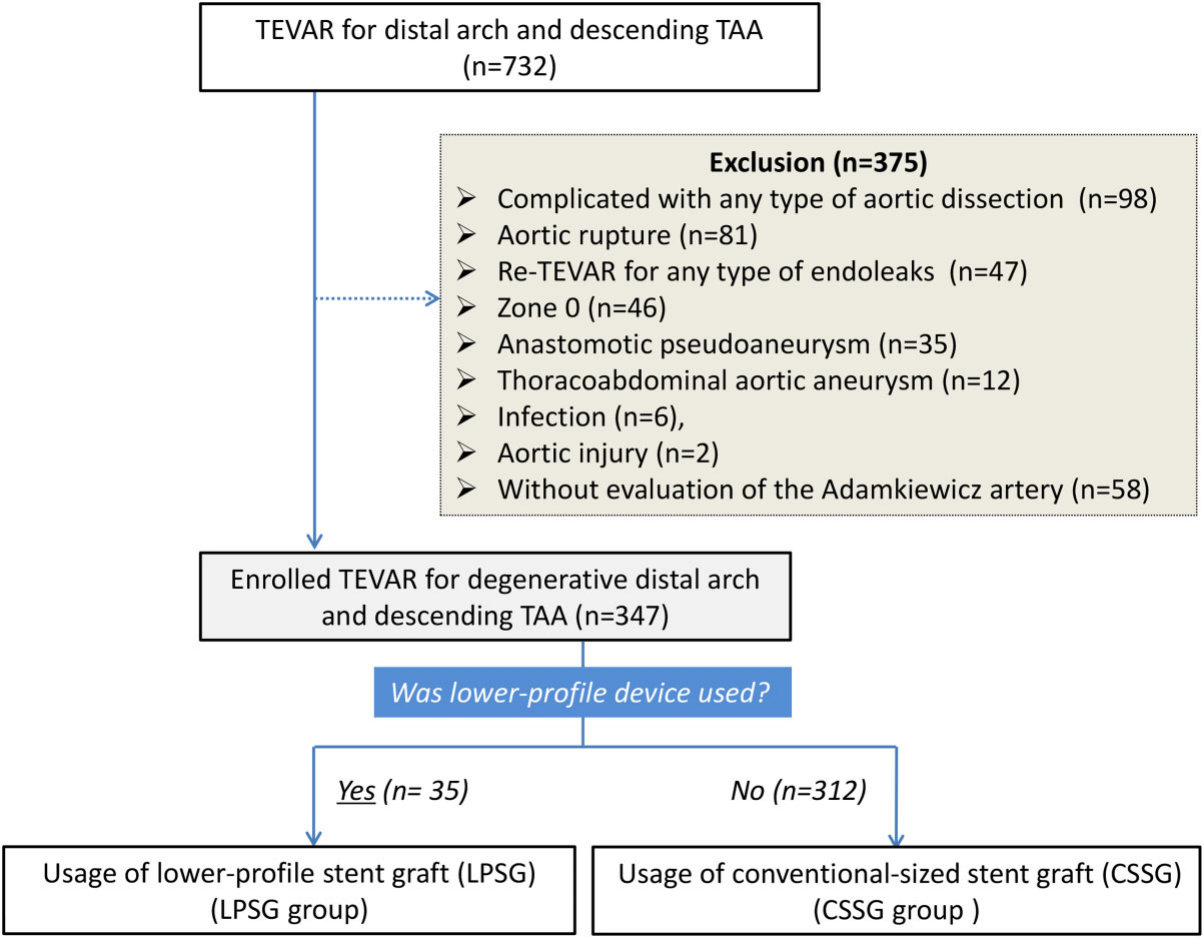
Flowchart of study population and method.

This observational, retrospective, cohort study was reported in line with the STROBE guidelines [8]. The patients, who underwent TEVAR with proximal landing from zone 1 to zone 4 for various descending TAA in our hospital from January 2009 to June 2021, were reviewed. Among these, patients with aortic dissection, aortic rupture, redo TEVAR for any type of endoleaks, proximal landing zone 0, anastomotic pseudoaneurysm, thoraco-abdominal aortic aneurysm, infection, aortic injury and without the Adamkiewicz artery (AKA) detection were excluded from the study (Fig. 1).

LPSG included Zenith Alpha (Cook Inc., Bloomington, USA), Valiant Navion (Medtronic, Inc., Minneapolis, MN, USA) and RELAY pro (Bolton Medical Inc., Sunrise, FL, USA). The conventional-sized stent graft (CSSG) included Gore TAG or cTAG (W.L. Gore and Assoc. Inc., Flagstaff, AZ, USA), Valiant Captivia (Medtronic, Inc., Minneapolis, MN, USA), Zenith TX2 (Cook Inc., Bloomington, USA) and RELAY Plus (Bolton Medical Inc., Sunrise, FL, USA).

### Evaluation of aortic wall thrombus

**Figure 2:**
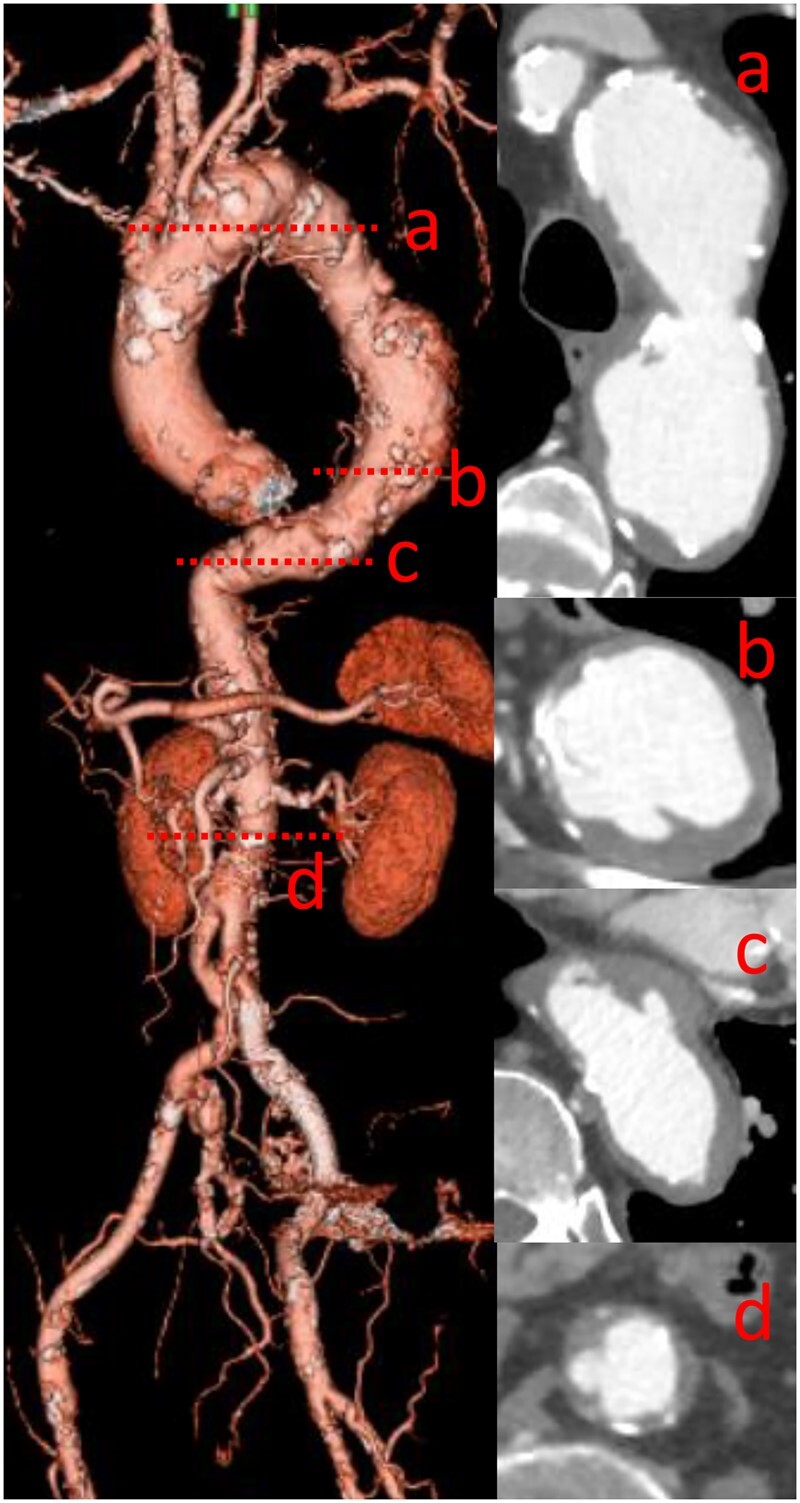
Shaggy aorta. The aortic images on computed tomography angiography of the patients with the typical case of shaggy aorta is showing; the red dotted lines from (a) to (d) indicate the location of each cross-section.

Qualitative evaluation of aortic wall thrombus was performed with computed tomography (CT) angiography and classified for aortic segments (limited to the thoracic aorta, limited to the abdominal aorta, or whole aorta), thrombus shape (smooth or irregular) and thickness (1–4 or >5 mm) [9]. Among them, the shaggy aorta was defined as an aortic intimal irregularity with an atheroma thickness of ≥5 mm in any aortic segment. This CT evaluation was performed by both a radiologist and a cardiovascular surgeon. Figure 2 shows a representative case of the shaggy aorta.

### Thoracic endovascular aortic repair techniques

TEVAR protocol have been standardized and remained the same over the course of the entire this study period and no specific medications including anticoagulant and/or statin were applied to the patients with shaggy aorta. Selection of the stent graft was individually made based on anatomical features and surgeon preferences. TEVAR procedures, including left subclavian artery (LSCA) and coeliac artery embolization, were performed by both aortic surgeons and interventional radiologists. The stent graft oversizing was set from 10% to 20% of the landing zone of the normal aorta by the manufacturer’s instructions for each device. A debranching bypass was performed to obtain an appropriate proximal landing zone length of >20 mm, and the details have been previously reported [6]. The right axillary artery was mainly applied as the inflow artery in zone 1 landing hybrid TEVAR and the left common carotid artery in zone 2 cases. An 8-mm expanded polytetrafluoroethylene graft was used for the bypass. The coeliac artery was closed to secure enough length of landing zone to prevent type Ib endoleak with or without the embolization to prevent type II endoleak.

Vascular access was basically through 1 side with direct surgical femoral exposure. Iliac access was considered in patients with a small external iliac artery diameter narrower than 7 mm and a past-history of bifurcated graft replacement of abdominal aortic aneurysm that may cause graft limb kinking and twisting. Preventative cerebrospinal fluid drainage starting intracranial pressure of <12 cm H_2_O was indicated in the patient with planned coverage of the intercostal artery branching AKA, extensive coverage of the descending aorta (the length of aortic coverage >300 mm or covered vertebrae >8), shaggy aorta, occluded LSCA and/or hypogastric artery and these combinations. To detect the location of the AKA, magnetic resonance angiography was applied at the beginning of the study period. Since 2011, CT angiography with a 16- or 64-channel multidetector-row helical CT scanner has been applied. The scan covered the area from the 7th thoracic vertebra to the 3rd lumbar vertebra and the rate of detection have been reported about 95% [10]. Procedures were performed under general anaesthesia, and motor-evoked potentials monitoring was made to perceive the possibility of spinal cord injury (SCI) in all patients [11]. The mean blood pressure was raised over 90 mmHg with the aggressive usage of catecholamines if necessary, in patients who had a significant change in motor-evoked potentials [12].

### Preventions for embolism

The use of balloon protection of the LSCA was applied to prevent embolic stroke mainly from the left vertebral artery in patients with proximal landing zones 1 and 2 since 2012. Details regarding its methods and value have been previously reported [6]. Aggressive hypotension using right ventricle rapid pacing was performed as additional preventive procedure to obtain secure stent graft deployment and reduce friction to the aortic arch wall. Carbon dioxide flushing before the saline flush was applied through the side port of the flushing chamber in TEVAR having a delivery sheath system, excluding cTAG (W.L. Gore and Assoc. Inc., Flagstaff, AZ, USA), to minimize the volume of air released during stent graft deployment since 2018 [13].

### Definitions and outcomes

LPSGs were defined as those marketed as thinner devices, which in this study included Zenith Alpha, Valiant Navion and RELAY pro. Embolic events were defined as stroke, SCI and other distal organ embolisms. Stroke was defined as any transient or permanent neurological dysfunction confirmed by a brain imaging test. SCI was defined as any transient or permanent neurological dysfunction in the lower extremity with or without bladder and bowel dysfunctions confirmed by magnetic resonance imaging. The diagnosis of stroke and SCI were made by a neurologist and transient ischaemic attack without any diagnostic imaging was not included. Distal embolism included ischaemic pancreatitis, ischaemic colitis, an irreversible worsening of renal function with elevated creatinine of >1 mg/dl and blue toe syndrome, whose origins were confirmed by contrast-enhanced CT.

The primary outcomes were the occurrence of any embolic events between the LPSG and the CSSG groups. The secondary outcomes included the incidence of hospital mortality, any type of immediate postoperative endoleaks and reintervention under the same hospitalization between the LPSG and the CSSG groups.

### Statistical analysis

Statistical analysis was conducted using Statistical Package for the Social Sciences for Windows, version 24.0 (SPSS Inc., Chicago, IL, USA). Categorical data were compared using Fisher’s exact test. Continuous variables were expressed as mean (standard deviation) and compared with the test after checking their normality by the Shapiro–Wilk test. The other non-normally distributed continuous variables were stated as median and compared by the Mann–Whitney *U* test. *P*-values of <0.05 were considered statistically significant. Univariate analysis was made by the logistic regression analysis to evaluate the influences of the covariables listed in Table 1 on embolic events. The primary and secondary outcomes were reassessed by propensity score matching (PSM). A logistic regression analysis was performed to calculate propensity scores for patients with embolic events using their baseline characteristics. We performed 1:1 PSM based on the nearest neighbour matching algorithm with replacement. The calliper width was set at 20% of the standard deviation of the propensity scores. In the subgroup analysis, *P*-value was adjusted by the conservative Bonferroni correction method. A Bonferroni-corrected *P*-value <0.025 was considered statistically significant [14].

**Table 1:** Patients’ characteristics and anatomical features (entire and matched cohorts)

Variable	Overall			Matched		
	Lower-profile stent graft (35)	Conventional-sized stent graft (312)	*P*-Value	Lower-profile stent graft (30)	Conventional-sized stent graft (30)	*P-*Value
Age (years), mean (SD)	77.0 (9.2)	77.0 (7.4)	0.994	76.9 (9.4)	79.2 (6.3)	0.514
Female sex, *n* (%)	8 (23)	65 (21)	0.827	6 (20)	7 (23)	1.000
SVS clinical comorbidity score, median (IQR)	4 (3–7)	5 (3–6)	0.652	5 (3–7)	5 (4–7)	0.358
Stroke history, *n* (%)	13 (37)	89 (29)	0.328	11 (37)	14 (47)	0.601
Chronic kidney disease (Cr > 1.5), *n* (%)	10 (29)	61 (20)	0.267	8 (27)	4 (13)	0.333
Coronary artery disease, *n* (%)	15 (43)	99 (32)	0.188	12 (30)	5 (17)	0.084
Respiratory disorder, *n* (%)	5 (14)	47 (15)	0.903	5 (17)	6 (20)	1.000
Antiplatelet therapy, *n* (%)	14 (40)	150 (48)	0.364	13 (43)	19 (63)	0.195
Anticoagulant therapy, *n* (%)	9 (26)	43 (14)	0.061	8 (27)	10 (33)	0.779
Shaggy aorta, *n* (%)	12 (34)	56 (20)	0.040	8 (27)	14 (47)	0.180
Occluded left subclavian artery, *n* (%)	0 (0)	3 (1.0)	0.560	0 (0)	0 (0)	N/A
Occluded unilateral hypogastric artery, *n* (%)	3 (8.6)	38 (12)	0.531	3 (10)	4 (13)	1.000
Prior aortic arch surgery, *n* (%)	5 (14)	66 (21)	0.340	5 (17)	0 (0)	0.052
Prior intervention on AAA, *n* (%)	8 (23)	59 (29)	0.575	6 (20)	2 (6.7)	0.254
Preoperative CSFD, *n* (%)	12 (34)	83 (27)	0.334	8 (27)	3 (10)	0.181
Iliac access, *n* (%)	0 (0)	78 (25)	<0.001	0 (0)	1 (3.3)	1.000
Proximal landing zone, median (IQR)	3 (2–4)	6 (5–8)	0.969	3 (1–4)	1 (1–2)	<0.001
Distal landing zone, median (IQR)	9 (6–11)	10 (7–11)	0.481	9 (6–10)	6 (5–9)	0.005
Zones covered by TEVAR, median (IQR)	6 (4–7)	6 (5–8)	0.803	6 (4–7)	4 (2–6)	0.008
Coverage of the ICA-AKA, *n* (%)	13 (37)	83 (27)	0.231	9 (30)	1 (3.3)	0.012
Coverage of the celiac trunk, *n* (%)	0 (0)	11 (3.5)	0.325	0 (0)	0 (0)	N/A
Outer diameter of the device (Fr), median (IQR)	22 (20–24)	24 (22–24)	0.028	–	–	–

AAA: abdominal aortic aneurysm; AKA: Adamkiewicz artery; Cr: creatinine; CSFD: cerebrospinal fluid drainage; ICA: intercostal artery; IQR: interquartile range; N/A: not applicable; SD: standard deviation; SVS: Society for Vascular Surgery; TEVAR: thoracic endovascular aortic repair.

## RESULTS

### Patients, baseline demographic and aneurysm characteristics

From the total of 732 patients, depending on the exclusion criteria (Fig. 1), the 347 patients were included in this study, and 35 for whom LPSG was used were assigned to the LPSG group (8 females; age: 77 ± 9.2 years), and the other 312 were assigned to the CSSG group (65 female; age: 77 ± 7.4 years) (Fig. 1). The patient features of each group are listed in Table 1. Society for Vascular Surgery clinical comorbidity score [15] revealed no difference between the 2 groups [4 (3–7) in LPSG vs 5 (3–6) in CSSG, *P *=* *0.652]. The LPSG group had a significantly higher prevalence of the shaggy aorta (34%) compared to the CSSG group (20%) (*P *=* *0.040). Iliac access was only applied for patients in the CSSG group (20%) and none in the LPSG group (0%) (*P *=* *0.040). No other difference was found in other variables (Table 1). Stentgrafts used in each group are described in Supplementary Material, Appendix S1.

### Early outcomes

A comparison of postoperative early mortality and complications during hospitalization is listed in Table 2. No 30-day mortality was observed in both groups. No difference was found in-hospital mortality between the 2 groups (2.9% [1/35] for LPSG vs 1.6% [5/312] for CSSG, *P *=* *0.589).

**Table 2:** Comparison of postoperative early mortality and complications during hospitalization (entire and matched cohorts)

Variable	Overall			Matched		
	Lower-profile stent graft (35)	Conventional-sized stent graft (312)	*P-*Value	Lower-profile stent graft (30)	Conventional-sized stent graft (30)	*P*-Value
30-Day mortality, *n* (%)	0 (0)	0 (0)	N/A	0 (0)	0 (0)	N/A
Hospital mortality, *n* (%)	1 (2.9)	5 (1.6)	0.589	1 (3.3)	1 (3.3)	1.000
Causes of hospital mortality						
Pneumonia	1	1	–	1	0	–
Suffocation	0	1	–	0	0	–
Infective endocarditis	0	1	–	0	0	–
Shower embolism	0	1	–	0	1	–
Peritonitis	0	1	–	0	0	–
Embolic complications						
Total numbers patients with embolic events, *n* (%)	0 (0)	36 (12)	0.034	0 (0)	9 (30)	0.002
Stroke, *n* (%)	0 (0)	8 (2.6)	0.338	0 (0)	4 (13)	0.112
Spinal cord ischaemia, *n* (%)	0 (0)	23 (7.4)	0.096	0 (0)	4 (13)	0.112
Distal embolism, *n* (%)	0 (0)	7 (2.6)	0.371	0 (0)	3 (10)	0.237
Ischaemic pancreatitis	0	2	–	0	1	–
Ischaemic colitis	0	2	–	0	1	–
Irreversible renal dysfunction	0	1	–	0	0	–
Blue toe syndrome	0	2	–	0	1	–
Hospital mortality + embolism, *n* (%)	1 (2.9)	39 (13)	0.090	1 (3.3)	9 (30)	0.012
Other complications, *n* (%)						
Stanford type A aortic dissection	0 (0)	0 (0)	N/A	0 (0)	0 (0)	N/A
Stanford type B aortic dissection	0 (0)	2 (0.6)	0.635	0 (0)	0 (0)	N/A
Pneumonia	2 (5.7)	7 (2.6)	0.221	2 (6.7)	0 (0)	0.492
Access route injury	1 (2.9)	3 (1.0)	0.319	1 (3.3)	0 (0)	1.000
Surgical site haematoma removal	0 (0)	6 (1.9)	0.408	0 (0)	0 (0)	N/A
Endoleaks at hospital discharge, *n* (%)						
Immediate type I endoleak	1 (2.9)	23 (7.4)	0.316	1 (3.3)	7 (23)	0.052
Immediate type II endoleak	1 (2.9)	29 (9.3)	0.199	1 (3.3)	4 (13)	0.353
Immediate type III endoleak	1 (2.9)	3 (1.0)	0.319	1 (3.3)	0 (0)	1.000

N/A: not applicable.

No retrograde type A aortic dissection was observed in both groups. No differences were found between the 2 groups (LPSG versus CSSG) in occurrences of postoperative complications, including Stanford type B dissection (0/35; 0% vs 2/312; 0.6%, *P *=* *0.635), pneumonia (2/35; 5.7% vs 7/312; 2.6%, *P* = 0.221), access route injury (1/35; 2.9% vs 3/312; 1.0%, *P *=* *0.319) and surgical site haematoma (0/35; 0% vs 6/312; 1.9%, *P *=* *0.408). CT scan revealed no differences between the 2 groups (LPSG vs CSSG) in incidences of immediate postoperative type I endoleak (1/35; 2.9% vs 23/312; 7.4%, *P *=* *0.316), type II (1/35; 2.9% vs 29/312; 9.3%, *P *=* *0.199) and type III (1/35; 2.9% vs 3/312; 1.0%, *P *=* *0.319) (Table 2).

### Embolic complications

Embolic events (LPSG/CSSG) included 0 (0%)/8 (2.6%) (*P *=* *0.715) for stroke, 0 (0%)/23 (7.4%) (*P* = 0.148) for SCI and 0 (0%)/8 (2.2%) (*P *=* *0.886) for other organ ischaemia, respectively. No difference was found in the frequency of embolism by the organs between the 2 groups. Distal embolism as other organ ischaemia in the CSSG group comprised ischaemic pancreatitis in 2 patients, ischaemic colitis in 2, irreversible renal dysfunction due to renal infarction in 1 and blue toe syndrome in 2. The total rate of patients with embolic events was significantly lower in LPSG (0/35 [0%]) than in CSSG (34/312 [11.2%]) (*P *=* *0.035) (Table 2).

### Analysis of matched cohorts by propensity score matching

After matching, 30 patients were selected from each group using the propensity scores to adjust for the risks for embolism including the frequency of age, shaggy aorta and iliac access as the variables related to embolic event (Table 1). PSM yielded the lower total rate of patients with embolic events in matched LPSG (0/30 [0%]) than in matched CSSG (9/30 [30.0%]) (*P *=* *0.002). The overall hospital mortality rate [1 (3.3%) vs 1 (3.3%); *P *=* *1.000] and the incidences of other perioperative complications (including immediate postoperative endoleaks and reintervention under the same hospitalization) did not differ between the study groups (Table 2).

### Risk factors for embolism

Age [odds ratio (OR): 1.005; *P *=* *0.040] and shaggy aorta (OR: 5.220; *P *<* *0.001) were identified as positive embolic event predictors. Other clinically noteworthy confounders included operative year (OR: 0.942, *P *=* *0.098), stroke (OR: 0.781, *P* = 0.542), chronic kidney disease (creatinine of >1.5) (OR: 1.576, *P *=* *0.254), after arch surgery (OR: 0.599, *P *=* *0.306), after abdominal aortic aneurysm repair (OR: 0.820, *P *=* *0.672) and zones of stent graft coverage (OR: 1.030, *P *=* *0.727) were not significant. Larger diameter of the device tended to be a risk of embolism (OR: 1.257, *P *=* *0.055) (Table 3).

**Table 3:** Univariate analysis: predictors of thromboembolic events during thoracic endovascular aortic repair

Covariate	OR	95% CI	*P-*Value
Operative year (2008–2021)	0.924	0.842–1.015	0.098
Mean age (years)	1.005	1.002–1.11	0.040
Female sex	1.081	0.471–2.485	0.854
SVS clinical comorbidity score	0.882	0.747–1.042	0.141
Old cerebral infarction	0.781	0.354–1.726	0.542
Chronic kidney disease (Cr > 1.5)	1.576	0.722–3.443	0.254
Coronary artery disease	0.679	0.307–1.502	0.339
Respiratory disorder	1.427	0.590–3.453	0.431
Antiplatelet therapy	0.998	0.500–1.992	0.996
Anticoagulant therapy	0.960	0.335–2.449	0.846
Shaggy aorta	5.220	2.541–10.724	<0.001
Occluded left subclavian artery	<0.001	N/A-0.000	0.999
Occluded unilateral hypogastric artery	1.577	0.613–4.055	0.344
Prior aortic arch surgery	0.599	0.224–1.600	0.306
Prior intervention on AAA	0.820	0.327–2.057	0.672
Preoperative CSFD	1.189	0.561–2.522	0.652
Iliac access	1.602	0.750–3.423	0.223
Proximal landing zone	0.939	0.795–1.110	0.462
Distal landing zone	1.038	0.900–1.197	0.609
Zones covered by TEVAR	1.030	0.874–1.214	0.727
Coverage of the Celiac trunk	1.974	0.410–9.513	0.397
Outer diameter of the device (Fr)	1.257	0.995–1.588	0.055

AAA: abdominal aortic aneurysm; CI: confidence interval; Cr: creatinine; CSFD: cerebrospinal fluid drainage; OR: odds ratio; SVS: Society for Vascular Surgery; TEVAR: thoracic endovascular aortic repair; N/A: not applicable.

### A subanalysis of the incidence of embolic events according to the presence or absence of shaggy aorta

As the subanalysis to evaluate the association of shaggy aorta and LPSG, patients were divided into 2 groups according to the presence or absence of shaggy aorta, and the difference in the incidence of embolic events between LPSG and CSSG was reassessed. The rate of total embolic events in 68 patients with shaggy aorta (12 in LPSG and 56 in CSSG) was significantly lower in LPSG (0/12 [0%]) than CSSG (19/56 [34%]) (*P *=* *0.015) (The Bonferroni-corrected *P *=* *0.022). The rate of total embolic events in 279 patients with non-shaggy aorta (23 in LPSG/256 in CSSG) reveals no difference between the 2 groups [0 (0%) and 16 (6.3%)] (*P *=* *0.377) (the Bonferroni-corrected *P *=* *0.190) (Table 4).

**Table 4:** Comparison of postoperative embolic complications during hospitalization in the subgroup analysis

Variable	Lower-profile stent graft (35)	Conventional-sized stent graft (312)	*P-value*
Embolic complications in patients with shaggy aorta (*n* = 68)	Low-profile device (12)	Conventional-sized device (56)	
Total numbers patients with embolic events, *n* (%)	0 (0)	18 (31)	0.022
Stroke, *n* (%)	0 (0)	5 (8.9)	0.282
Spinal cord ischaemia, *n* (%)	0 (0)	12 (21)	0.094
Distal embolism, *n* (%)	0 (0)	4 (7.1)	0.340
Ischaemic pancreatitis	0	2	-
Ischaemic colitis	0	1	-
Blue toe syndrome	0	1	-
Embolic complications in patients with non-shaggy aorta (*n* = 279)	Lower-profile stent graft (23)	Conventional-sized stent graft (256)	
Total numbers patients with embolic events, *n* (%)	0 (0)	18 (12)	0.189
Stroke, *n* (%)	0 (0)	3 (1.2)	0.602
Spinal cord ischemia, *n* (%)	0 (0)	12 (7.0)	0.289
Distal embolism, *n* (%)	0 (0)	3 (1.2)	0.602
Ischaemic colitis	0	1	-
Irreversible renal dysfunction	0	1	-
Blue toe syndrome	0	1	-

## DISCUSSION

Several preventive measures for embolism have been previously reported [3–6]. We have applied the balloon protection of the LSCA and aggressive hypotension using right ventricular rapid pacing to reduce friction to the aortic arch wall during hybrid arch TEVAR [6], and carbon dioxide flushing technique to prevent air embolization in the usage of stent graft system with outer sheath [13]. However, we still encountered embolic events, and further reduction of atherosclerotic embolism was critical.

Among recent advancements in stent graft technology, LPSG might contribute to reducing embolization by less friction to the aortic wall during TEVAR compared with the CSSG. El Beyrouti *et al.* [16] reported the early results of TEVAR using RELAY pro devices, of which the delivery sheath is designed 3- to 4-Fr smaller in outer diameter than the previous-generation devices of RELAY Plus. Their study revealed no thromboembolic complications, including endograft thrombosis, obstruction, stroke and SCI. Riambau *et al.* [17] revealed only 1 stroke (3%) in a patient after TEVAR with proximal landing zone 0 and no SCI in a prospective, multicentre, single-arm regeneration study. Melissano *et al.* [18] provided midterm results in 262 patients using Zenith Alpha devices for various aortic pathologies that revealed SCI in 10 patients (3.7% of 270 procedures), and its incidence rate was significantly higher in patients with type B aortic dissection. This result was not due to thromboembolism, but rather to the contribution of dissection that involved intercostal arteries. Further, a greater stroke rate was associated with treated aortic arch lesions in zones 0 and 1 (13.3% vs 2.9% for the others, *P *=* *0.006). Especially, TEVAR with proximal landing zone 0 has a high risk of stroke [19], and our study did not include TEVAR with total debranching bypass to avoid this bias issue. Additionally, this study cohort in both groups was limited to the patients who were treated due to degradative TAA, excluding aortic pathologies of aortic dissection, aortic rupture, pseudoaneurysm, trauma, infection, thoraco-abdominal aortic aneurysm and re-TEVAR, to verify comparison in patients with and without LPSG.

Our study, which was limited to TEVAR for degenerative TAA, revealed no major neurological abnormalities or thromboembolic events in the LPSG group, and the rate of total embolic events, including stroke, SCI and other organ ischaemia, was significantly lower in the LPSG group (0%) than in the CSSG group (11.2%) (*P *=* *0.035). Our result suggested that LPSGs might reduce postoperative thromboembolic events compared with the CSSGs, although no similar comparison between LPSG and CSSG had been previously published.

Moreover, we examined how this result would change in the presence or absence of a shaggy aorta, which is the most important factor in causing atherosclerotic embolism during TEVAR [1, 20–22]. Our Logistic regression analysis highlighted shaggy aorta (OR: 5.220; *P *<* *0.001) as a positive predictor of embolic events in addition to elderly age in terms of the risk factor for thromboembolic events. With this result, the subanalysis was performed to equalize the different patient aortic wall conditions by the presence or absence of a shaggy aorta, and the advantage of the LPSGs was more emphasized in patients with severe atherosclerosis change in the aortic wall.

Besides, these differences in outcomes between the LPSG group and the CSSG may be due to the difference in frictional force generated between the aortic wall and the device, i.e., the difference in the contact area. Interestingly, Perera *et al.* [7] highlighted the significance of reducing contact with an atheromatous aortic arch wall, thereby reducing the dislodgement of particulate matter and resulting in less embolization. They applied intraoperative transcranial Doppler to detect the number of high-intensity transient signals as evidence of stroke. Additionally, they emphasized that transcranial Doppler mostly detected high-intensity transient signals with high frequency during stent graft deployment. Thus, these results support our hypothesis that the use of LPSG has an embolism-reducing effect by decreasing the frictional forces generated between the aortic wall and the device in patients with a shaggy aorta.

The aortic arch is noteworthy as a site of high friction because the resulting embolism is cerebral infarction, which is one of the most devastating thromboembolic events. Bismuth *et al.* revealed a significantly greater number of micro embolic signals by transcranial Doppler when the device was deployed more proximally. Furthermore, proximal landing zones 0–2 were associated with more embolic signals than zones 3 and 4 in the treatment phase encompassing device deployment [23]. Therefore, some devices were designed to reduce friction at the aortic arch by adding a curve to the device sheath. These efforts are based on the importance of reducing friction between the device and the aortic wall during TEVAR procedures and support our concept of reducing friction with the aorta by the LPSGs.

As for endoleaks, no reports were found to be associated with embolism. In this study, there was no difference in the incidence of endoleaks between the 2 groups, but the CSFG group had a higher rate [(LPSG vs CSSG) in incidences of type I endoleak (2.9% vs 7.4%, *P *=* *0.316) and type II (2.9% vs 9.3%, *P *=* *0.199)]. Especially, among type I endoleaks, 39% (9/23) were Ia endoleak, of which 7 were occurred in proximal zone 1 cases, likely influenced by the proximal landing zone.

### Limitations

First, this single-centre, retrospective observational, study on a specific cohort of patients was limited by sample size in the LPSG group. Additional investigations are mandatory to authorize these outcomes. Second, better-quality TEVAR techniques, including launched devices of LPSGs, and the selection on the type of stent graft might affect the outcomes. Third, asymptomatic embolisms, including stroke, might have been missed. Third, the impact of statin therapy has not been evaluated. Finally, some SCI diagnoses were made by clinical features without visualization using MRI due to technical issues.

## CONCLUSIONS

The use of LPSG could reduce embolism in TEVAR, and the difference was more pronounced in patients with a shaggy aorta. LPSG might be beneficial in preventing embolism in TEVAR for patients with a shaggy aorta.

## Supplementary Material

ivad058_Supplementary_Data

## Data Availability

The data underlying this article cannot be shared publicly because of relevant data protection regulations. However, the data will be shared on reasonable request to the corresponding author with permission from the ethics committee.

## Notes

Presented at the 36th Annual Meeting of the European Association for Cardio-Thoracic Surgery, Milan, Italy, 5–8 October 2022.

## ABBREVIATIONS

AKA: Adamkiewicz artery
CT: Computed tomography
CSSG: Conventional-sized stent graft
LPSG: Lower-profile stent graft
LSCA: Left subclavian artery
OR: Odds ratio
PSM: Propensity score matching
SCI: Spinal cord injury
TAA: Thoracic aortic aneurysm
TEVAR: Thoracic endovascular aortic repair
