# Supplementary material for: Lower-profile stent graft reduces the risk of embolism during thoracic endovascular aortic repair in shaggy aorta
Source: Interdiscip Cardiovasc Thorac Surg. 2023 Apr 24;36(5):ivad058. doi: 10.1093/icvts/ivad058 (PMC10191628; doi:10.1093/icvts/ivad058)
Supplement: ivad058_Supplementary_Data [file ivad058_supplementary_data.docx]

*Stentgrafts used in each group*

The Zenith Alpha was used in 15 (43%), Valiant Captivia and Valiant Navion in 14 (40%), and RELAY pro in 6 (17%) patients in the LPSG group. Gore TAG or cTAG was used in 150 (48%), Talent in 12 (3.8%), Valiant Captivia in 83 (27%), Zenith TX2 in 25 (8.2%), and RELAY Plus in 42 (13%) patients in the CSSG group.
